# SERKET: An Architecture for Connecting Stochastic Models to Realize a Large-Scale Cognitive Model

**DOI:** 10.3389/fnbot.2018.00025

**Published:** 2018-06-26

**Authors:** Tomoaki Nakamura, Takayuki Nagai, Tadahiro Taniguchi

**Affiliations:** ^1^Department of Mechanical Engineering and Intelligent Systems, University of Electro-Communications, Tokyo, Japan; ^2^Department of Information Science and Engineering, Ritsumeikan University, Shiga, Japan

**Keywords:** cognitive models, probabilistic generative models, symbol emergence in robotics, concept formation, unsupervised learning

## Abstract

To realize human-like robot intelligence, a large-scale cognitive architecture is required for robots to understand their environment through a variety of sensors with which they are equipped. In this paper, we propose a novel framework named Serket that enables the construction of a large-scale generative model and its inferences easily by connecting sub-modules to allow the robots to acquire various capabilities through interaction with their environment and others. We consider that large-scale cognitive models can be constructed by connecting smaller fundamental models hierarchically while maintaining their programmatic independence. Moreover, the connected modules are dependent on each other and their parameters must be optimized as a whole. Conventionally, the equations for parameter estimation have to be derived and implemented depending on the models. However, it has become harder to derive and implement equations of large-scale models. Thus, in this paper, we propose a parameter estimation method that communicates the minimum parameters between various modules while maintaining their programmatic independence. Therefore, Serket makes it easy to construct large-scale models and estimate their parameters via the connection of modules. Experimental results demonstrated that the model can be constructed by connecting modules, the parameters can be optimized as a whole, and they are comparable with the original models that we have proposed.

## 1. Introduction

To realize human-like robot intelligence, a large-scale cognitive architecture is required for robots to understand their environment through a variety of sensors with which they are equipped. In this paper, we propose a novel framework that enables the construction of a large-scale generative model and its inferences easily by connecting sub-modules in order for robots to acquire various capabilities through interactions with their environment and others. We consider it important for robots to understand the real world by learning from their environment and others, and have proposed a method that enables robots to acquire concepts and language (Nakamura et al., 2014; Attamimi et al., 2016; Nishihara et al., 2017; Taniguchi et al., 2017) based on the clustering of multimodal information that they obtain. These proposed models are based on Bayesian models with complex structures, and we derived and implemented the parameter estimation equations. If we realize a model that enables robots to learn more complicated capabilities, we have to construct a more complicated model, and derive and implement equations for parameter estimation. However, it is difficult to construct higher-level cognitive models by leveraging this approach. Alternatively, these models can be interpreted as a composition of more fundamental Bayesian models. In this paper, we develop a large-scale cognitive model by connecting the Bayesian models and propose an architecture named Serket (Symbol Emergence in Robotics tool KIT^1^), which enables the easier construction of such models.

In the field of cognitive science, cognitive architectures (Laird, 2008; Anderson, 2009) have been proposed to implement human cognitive mechanisms by describing human perception, judgment, and decision-making. However, complex machine learning algorithms have not yet been introduced, which makes it difficult to implement our proposed models. Serket makes it possible to implement more complex models by connecting modules.

One approach to develop a large-scale cognitive model is the use of probabilistic programming languages (PPLs), which make it easy to construct Bayesian models (Patil et al., 2010; Goodman et al., 2012; Wood et al., 2014; Carpenter et al., 2016; Tran et al., 2016). PPLs can construct Bayesian models by defining the dependencies between random variables, and the parameters are automatically estimated without having to derive the equations for them. By using PPLs, it is easy to construct relatively small-scale models, such as a Gaussian mixture model and latent Dirichlet allocation, but it is still difficult to model multimodal sensory information, such as images and speech obtained by the robots. Because of this, we implemented models for concept and language acquisition, which are relatively large-scale models, as standalone models without PPLs. However, we consider the approach where an entire model is implemented by itself has limitations if it is constructed as a large-scale model.

Large-scale cognitive models can be constructed by connecting smaller fundamental models hierarchically; in fact, our proposed models have such a structure. In the proposed novel architecture Serket, large-scale models were constructed by hierarchically connecting smaller-scale Bayesian models (hereafter, each one is referred to as a *module*) while maintaining their programmatic independence. The connected modules are dependent on each other, and parameters must be optimized as a whole. When models are constructed by themselves, the parameter estimation equations have to be derived and implemented depending on the models. However, in this paper, we propose a method for parameter estimation by communicating the minimum parameters between various modules while maintaining their programmatic independence. Therefore, Serket makes it easy to construct large-scale models and estimate their parameters by connecting modules.

In this paper, we propose the Serket framework and implement models that we proposed by leveraging this framework. Experimental results demonstrated that the model can be constructed by connecting modules, the parameters can be optimized as a whole, and they are comparable with original models that we have proposed.

## 2. Background

### 2.1. Symbol emergence in robotics

Recently, it has been said that artificial intelligence is superior to human intelligence in the area of supervised learning, as typified by deep learning as far as certain specific tasks (He et al., 2015; Silver et al., 2017). However, we believe that it is difficult to realize human-like intelligence only via supervised learning because all supervised labels cannot be obtained for all the sensory information of robots. To this end, we believe that it is also important for robots to understand the real environment by structuring their own sensory information in an unsupervised manner. We consider such a learning process as a symbol emergence system (Taniguchi et al., 2016a).

**Figure 1:**
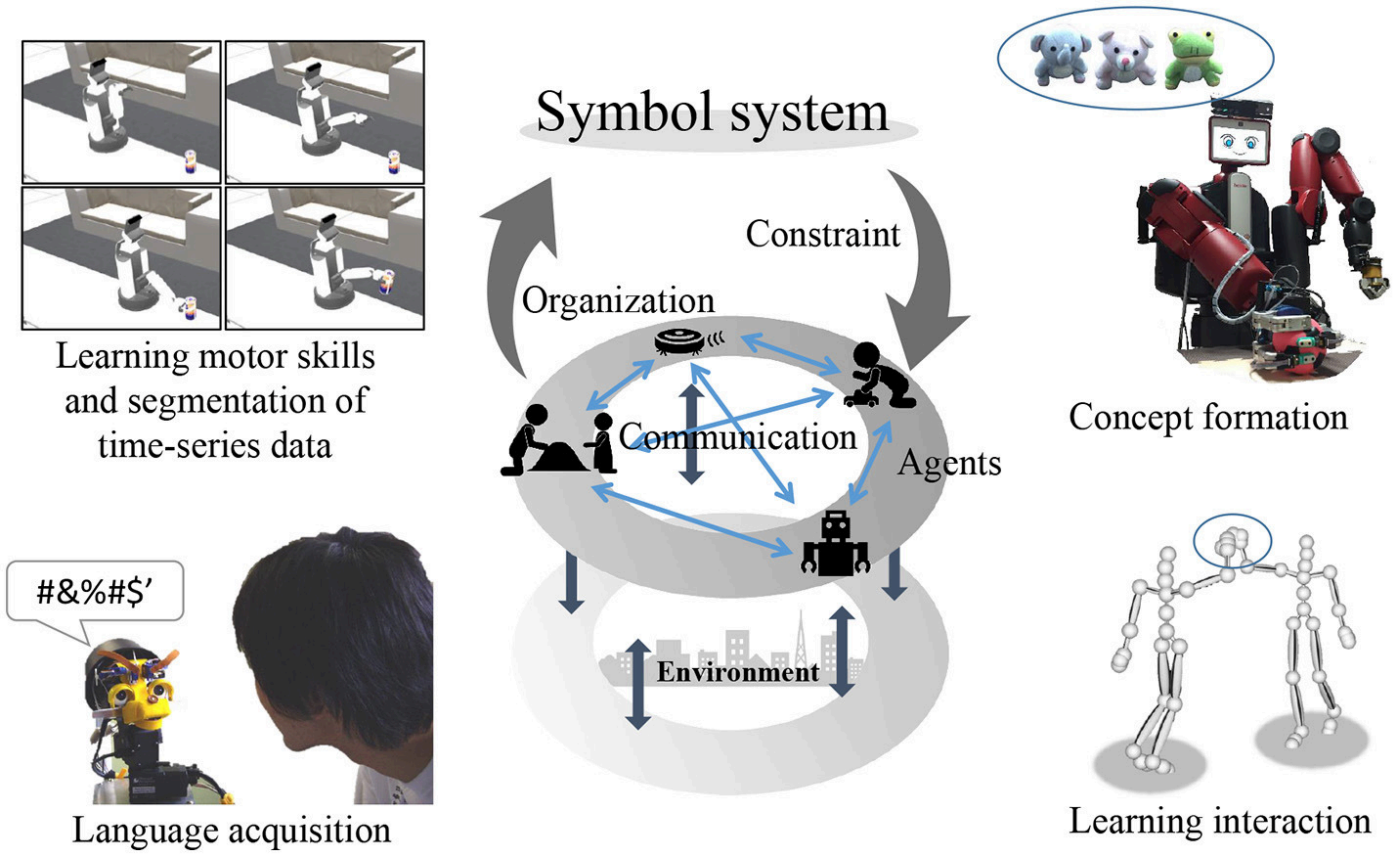
Symbol emergence system.

The symbol emergence system is based on the genetic epistemology proposed by Piaget (Piaget and Duckworth, 1970). In genetic epistemology, humans organize symbol systems in a bottom-up manner through interaction with the environment. Figure 1 presents an overview of the symbol emergence system. The symbols are self-organized from sensory information obtained through interactions with the environment. However, it can be difficult for robots to communicate with others using symbols learned only in a bottom-up manner, because the sensory information cannot be shared directly with others and the meaning of symbols differs depending on the individual. To communicate with others, the meanings of symbols must be transformed into common meanings among individuals through their interactions. This is considered as a top-down effect from symbols to individuals' organization of them. Thus, in the symbol emergence system, the symbols emerge through loops of top-down and bottom-up effects. In the symbol emergence in robotics, symbols include not only linguistic symbols but also various types of knowledge self-organized by robots. Therefore, symbol emergence in robotics covers a wide range of research topics, such as concept formation (Nakamura et al., 2007), language acquisition (Taniguchi et al., 2016b, 2017; Nishihara et al., 2017), learning of interactions (Taniguchi et al., 2010), learning of body schemes (Mimura et al., 2017), and learning of motor skills and segmentation of time-series data (Taniguchi et al., 2011; Nakamura et al., 2016).

We have proposed models that enable robots to acquire concepts and language by considering its learning process as a symbol emergence system. The robots form concepts in a bottom-up manner, and acquire word meanings by connecting words and concepts. Simultaneously, words are shared with others, and their meanings are changed through communication with others. Therefore, such words affect concept formation in a top-down manner, and concepts are changed. Thus, we have considered that robots can acquire concepts and word meanings through loops of bottom-up and top-down effects.

### 2.2. Existing cognitive architecture

There have been many attempts to develop intelligent systems. In the field of cognitive science, cognitive architectures (Laird, 2008; Anderson, 2009) have been proposed to implement humans cognitive mechanisms by describing human perception, judgment, and decision-making. As mentioned earlier, it is important to consider how to model the multimodal sensory information obtained by robots. However, this is still difficult to achieve with these cognitive architectures. To construct more complex models, some frameworks have been proposed in the field of machine learning.

Frameworks of deep neural networks (DNNs) such as TensorFlow (Abadi et al., 2016), Keras (Chollet, 2015), and Chainer (Tokui et al., 2015) have been developed. These frameworks make it possible to construct DNN models and estimate their parameters easily. These frameworks are one of the reasons why DNNs have been widely used for several years.

Alternatively, PPLs that make it easy to construct Bayesian models have also been proposed (Patil et al., 2010; Goodman et al., 2012; Wood et al., 2014; Carpenter et al., 2016; Tran et al., 2016). The advantages of PPLs are that they can construct Bayesian models by defining the dependencies between random variables, and the parameters are automatically estimated without deriving equations for them. By using PPLs, relatively small-scale models, such as the Gaussian mixture model and latent Dirichlet allocation (LDA), can be constructed easily. However, it is still difficult to model multimodal sensory information, such as images and speech obtained by the robots. We believe that a framework by which a large-scale probabilistic generative model can be more easily constructed is required to model the multimodal information of the robot.

### 2.3. Cognitive architecture based on probabilistic generative model

**Figure 2:**
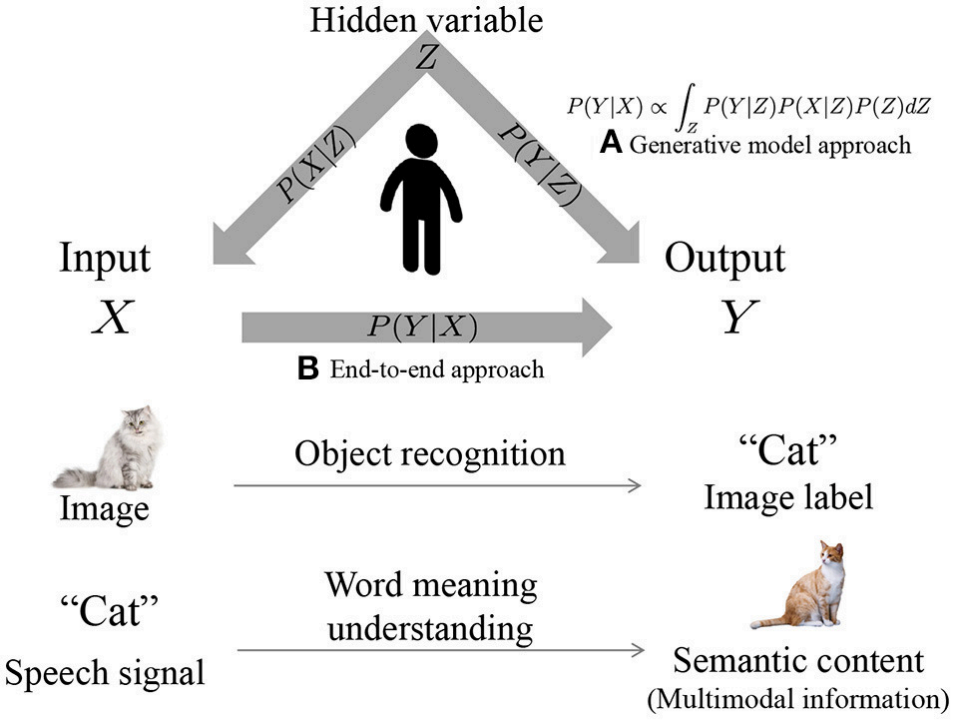
Overview of cognitive model by **(A)** probabilistic generative model and **(B)** end-to-end learning.

We believe that cognitive models make it possible to predict an output *Y* against an input *X*. For example, as shown in Figure 2, an object label *Y* is predicted from a sensor input *X* via object recognition. It is through the understanding of word meanings that the semantic content *Y* are predicted from speech signal *X*. In other words, the problem can be defined as how to model *P*(*Y*|*X*), where the prediction is realized by argmax_*Y*_*P*(*Y*|*X*). DNNs model relationships between an input *X* and output *Y* directly by an end-to-end approach (Figure 2B). Alternatively, we considered developing these cognitive models by leveraging Bayesian models, where *X* and *Y* are treated as random variables, and the relationships between them are represented by a latent variable *Z* (Figure 2A). Therefore, in Bayesian models, the prediction of output *Y* from input *X* is computed as follows:

(1) $$\begin{array}{l} P\left(Y|X\right)\propto P\left(Y,X\right) \end{array}$$

(2) $$\begin{array}{l} =\underset{Z}{\int }P\left(Y|Z\right)P\left(X|Z\right)P\left(Z\right)dZ. \end{array}$$

**Figure 3:**
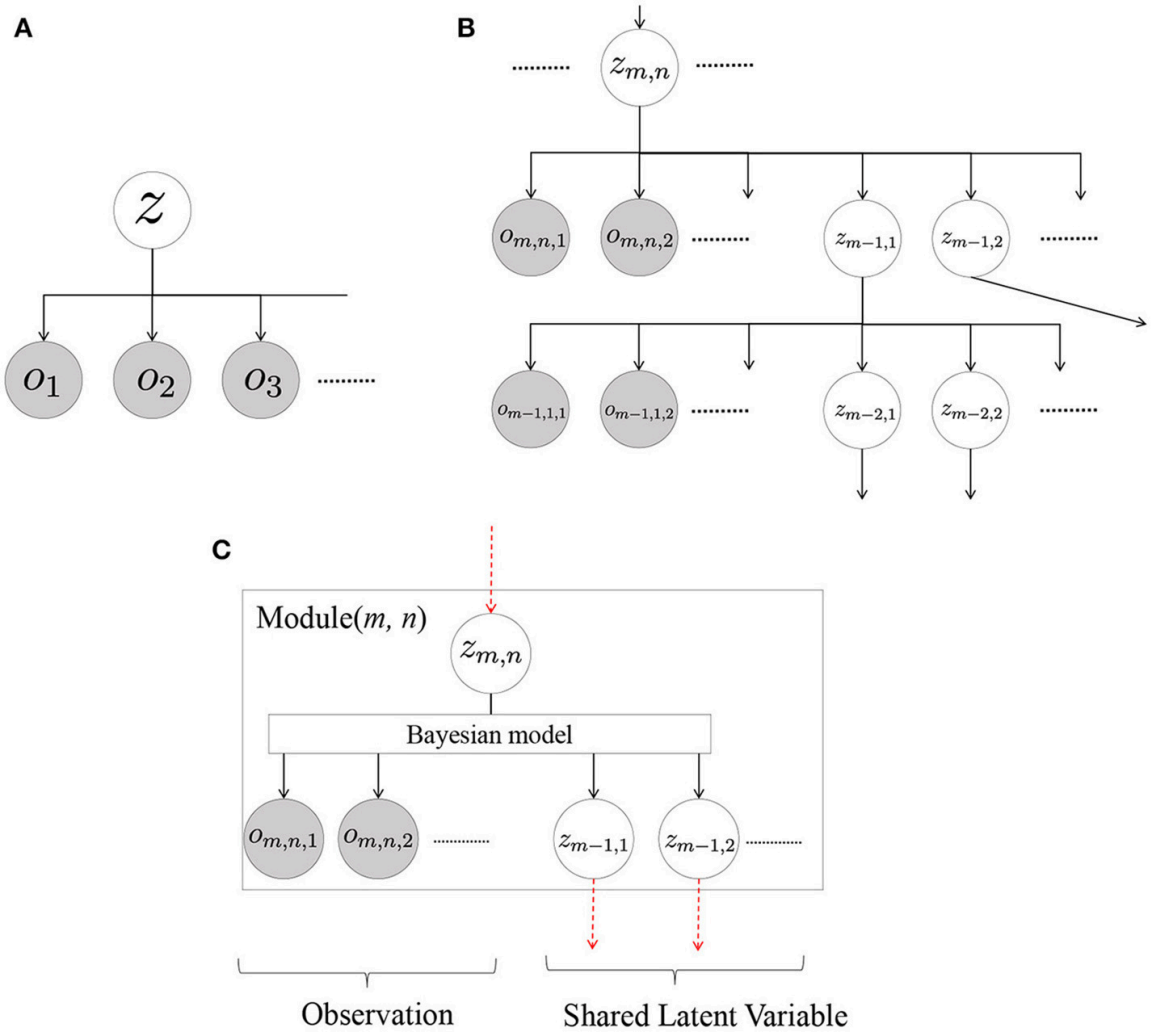
Generalized hierarchical cognitive model: **(A)** single-layer model, **(B)** multilayered model by hierarchicalization of single-layer models, and **(C)** generalized form of a module in Serket.

This is multimodal latent Dirichlet allocation (MLDA) (Blei and Jordan, 2003; Nakamura et al., 2009; Putthividhy et al., 2010), the details of which are described in the Appendix. However, MLDA is based on the important assumption that the observed variables *X* and *Y* are conditionally independent against latent variable *Z*. Here, we consider models where assumptions are made about multiple observations without distinguishing between input and output. Figure 3A displays the generalized model, where the right side of Equation (1) corresponds to the following equation, and a part of the observations can be predicted from other observations.

(3) $$\begin{array}{l} P\left(o_{1},o_{2},\cdots \;\right)=\int_{z}P\left(z\right)\Pi_{n}P\left(o_{n}|z\right)dz. \end{array}$$

As mentioned earlier, it is assumed that all observations ***o***_1_, ***o***_2_, ⋯  are conditionally independent against *z*. This assumption is often used to deal with multimodal data (Blei and Jordan, 2003; Wang et al., 2009; Putthividhy et al., 2010; Françoise et al., 2013) because modeling all dependencies makes parameter estimation difficult.

Considering the modeling of various sensor data as observations ***o***_1_, ***o***_2_, ⋯ , it is not always true for all the observations to satisfy the conditionally independent assumption. In general, the information surrounding us has a hierarchical structure. Hence, a hierarchical model can be used to avoid this difficulty (Attamimi et al., 2016). Furthermore, latent variables, such as concepts, are generally related to each other, and such relationships can be represented by hierarchical models. Figure 3B represents a hierarchical version of Figure 3A and can be thought of as generalization of the cognitive architecture based on a probabilistic generative model. It should be noted that the structure can be designed manually (Attamimi et al., 2016) and/or found autonomously by using a structure learning method (Margaritis, 2003), which is beyond the scope of this paper. In this hierarchized model, o_*, *_ are observations and z_*, *_ are latent variables, and the right side of Equation (1) corresponds to the following equation:

(4) $$\begin{array}{l} P\left(O|z_{M,1},z_{M,2},\cdots \;\right)=\overset{M}{\underset{m}{\prod }}\overset{{\overset{-}{N}}_{m}}{\underset{n}{\prod }}\int_{z_{m,n}}P\left(z_{m,n}\right)\overset{N_{m}}{\underset{i}{\prod }}P\left(o_{m,n,i}|z_{m,n}\right)\\ \overset{{\overset{-}{N}}_{m-1}}{\underset{n^{\prime }}{\prod }}P\left(z_{m-1,n^{\prime }}|z_{m,n}\right)dz_{m,n}, \end{array}$$

where ***O*** is the set of all observations, *M* is the number of the hierarchy, and *N*_*m*_ and ${\overset{-}{N}}_{m}$ denote the number of observations and latent variables in the *m*-th hierarchy, respectively. In this model, it is not difficult to analytically derive equations to estimate the parameters if the number of the hierarchy is not large. However, it is more difficult to derive them if the number of the hierarchy increases. To estimate the parameters of the hierarchical model, we propose Serket, which is an architecture that renders it possible to estimate the parameters by dividing them into even hierarchies.

From the viewpoint of hierarchical models, many studies have proposed models that capture the hierarchical nature of the data (Li and McCallum, 2006; Blei et al., 2010; Ghahramani et al., 2010; Ando et al., 2013; Nguyen et al., 2014). On the other hand, Serket models the hierarchical structure of modalities. For such hierarchical models, methods based on LDA (Li et al., 2011; Yang et al., 2014) have been proposed, and we have also proposed multilayered MLDA (Attamimi et al., 2016). These models are the simplest examples constructed by Serket. In this paper, we construct these models by dividing them into smaller modules.

### 2.4. Cognitive models

In the past, studies on how the relationships between multimodal information are modeled have been conducted (Roy and Pentland, 2002; Wermter et al., 2004; Ridge et al., 2010; Ogata et al., 2010; Lallee and Dominey, 2013; Zhang et al., 2017). Neural networks were used in these studies, which made inferences based on observed information possible by learning multimodal information, such as words, visual information, and a robot's motions. As mentioned earlier, these are some examples of the cognitive models that we defined.

There are also studies in which manifold learning was used for modeling a robot's multimodal information (Mangin and Oudeyer, 2013; Yuruten et al., 2013; Mangin et al., 2015; Chen and Filliat, 2015). These studies used manifold learning such as non-negative matrix factorization, in which multimodal information is represented by low-dimensional hidden parameters. We consider this as another approach to constructing cognitive models, in which the information is inferred through hidden parameters.

Recently, DNNs have made notable advances in many areas such as object recognition (He et al., 2015), object detection (Redmon et al., 2016), speech recognition (Amodei et al., 2016), sentence generation (Vinyals et al., 2015), machine translation (Sutskever et al., 2014), and visual question answering (Wu et al., 2016). In these studies, end-to-end learning was used, which made it possible to infer information from other information. Therefore, these are also considered part of the cognitive model defined in this paper. However, as mentioned in section 2.1, we believe that it is important for robots to understand the real environment by structuring their own sensory information in an unsupervised manner.

**Figure 4:**
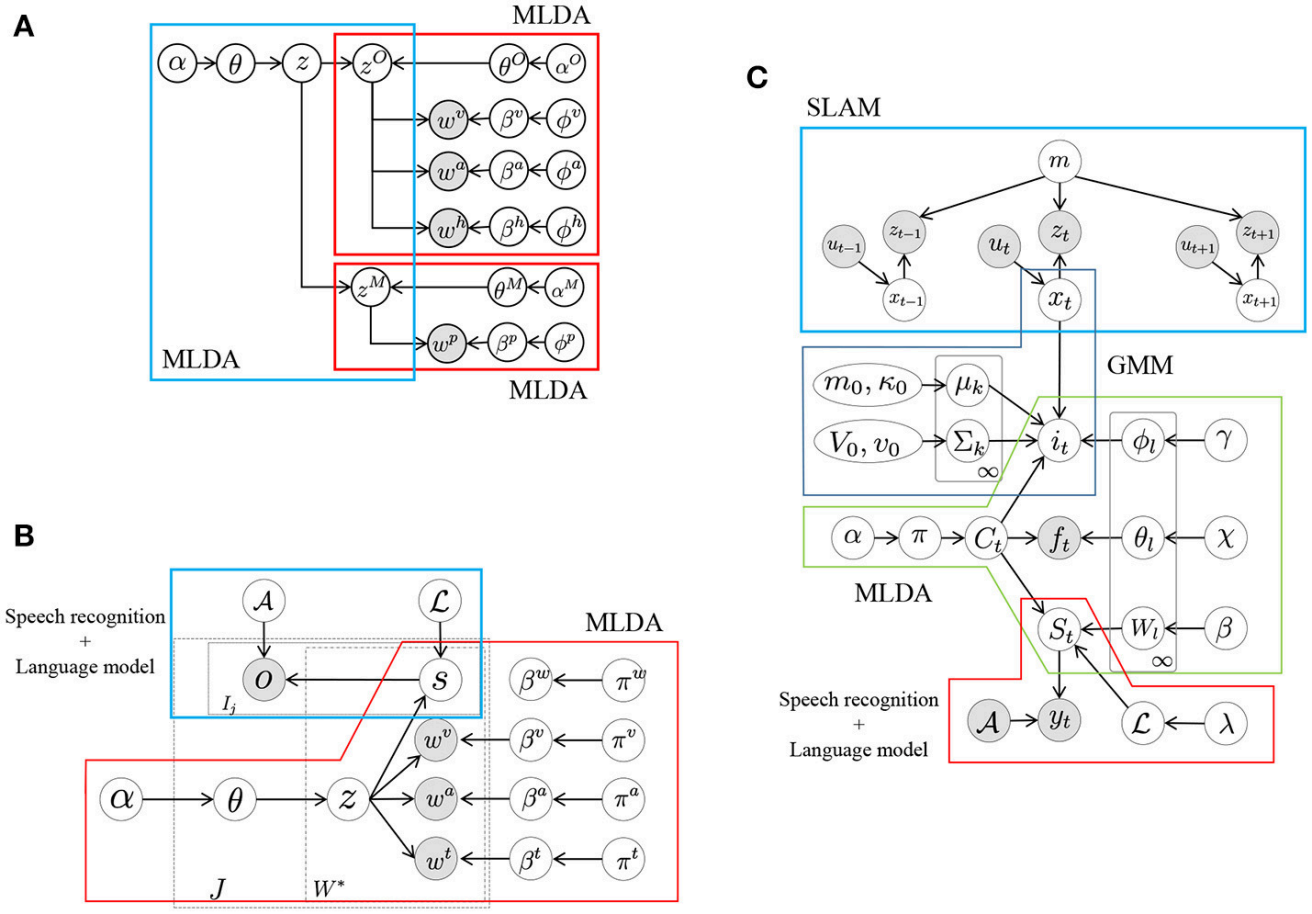
Graphical models for concept formation: **(A)** model for hierarchical concept (Attamimi et al., 2016) constructed with multimodal latent Dirichlet allocations (MLDAs), **(B)** model for object concept and language acquisition (Nakamura et al., 2014; Nishihara et al., 2017) constructed with MLDAs and speech recognition, and **(C)** model for location concept and language acquisition (Taniguchi et al., 2017) constructed with simultaneous localization and mapping (SLAM), Gaussian mixture model (GMM), MLDA, and speech recognition.

To develop a cognitive model where robots learn autonomously, our group proposed several models for concept formation (Nakamura et al., 2007), language acquisition (Taniguchi et al., 2016b, 2017; Nishihara et al., 2017), learning of interactions (Taniguchi et al., 2010), learning of body schemes (Mimura et al., 2017), learning motor skills, and segmentation of time series data (Taniguchi et al., 2011; Nakamura et al., 2016). Although all of these are targets of Serket, we focused on concept formation in this paper. We define concepts as categories into which the sensory information is classified, and propose various concept models. These are implementations of the aforementioned hierarchical model. Figure 4A displays one of our proposed models. This is the simplest form of the hierarchical model, where *z*^*O*^ and *z*^*M*^ denote an object and a motion concept, respectively, and their relationship is represented by *z* (Attamimi et al., 2016). Therefore, in this model, *z* represents objects and possible motions against them, which are considered as their usage, and observations become conditionally independent by introducing the latent variables *z*^*O*^ and *z*^*M*^.

In these Bayesian models, the latent variables shown as the white nodes *z, z*^*O*^, and *z*^*M*^ in Figure 4A can be learned from the observations shown as gray nodes in an unsupervised manner. Moreover, these latent variables are not determined independently but optimized as a whole by depending on each other. Although it seems that this model has a complex structure and that it is difficult to estimate the parameters and determine the latent variables, this model can be divided into smaller components, each of which is an MLDA model. The models shown in Figures 4B,C can also be divided into smaller components despite their complex structure. Similar to these models, it is possible to develop larger models by combining smaller models as modules. In this paper, we propose a novel architecture Serket to develop larger models by combining modules.

In the proposed architecture, the parameters of each module are not learned independently but learned based on their dependence on each other. To implement such learning, it is important to share latent variables between modules. For example, *z*^*O*^ and *z*^*M*^ are shared between two MLDAs in the model, respectively, as shown in Figure 4A. The shared latent variables were not determined independently but determined depending on each other. Serket makes it possible for each module to maintain its independence as a program as well as be learned as a whole through the shared latent variables.

## 3. Serket

### 3.1. Composing cognitive sub-modules

Figure 3C displays the generalized form of the module assumed in Serket. In this figure, we omit the detailed parameters for generalization because we assume that any type of models can be the modules of Serket. Each module has multiple shared latent variables *z*_*m*−1, *_ and observations ***o***_*m, n*, *_, which are assumed to be generated from latent variable *z*_*m, n*_ of a higher level. Modules with no shared latent variable or observations are also included in the generalized model. Moreover, the modules can have any internal structure as long as they have shared latent, observation, and higher-level latent variables. Based on this module, a larger model can be constructed by connecting the latent variables of module(*m*−1, 1), module(*m*−1, 2), ⋯  recursively. In the Serket architecture, each module must satisfy the following requirements:

1. In each module with shared latent variables, the probability that latent variables are generated can be computed as
  (5) $$\begin{array}{l} P\left(z_{m-1,i}|z_{m,n},o_{m,n,1},o_{m,n,2},\cdots \;,z_{m-1}\right). \end{array}$$
2. The module can send the following probability by leveraging one of the methods explained in the next section:
  (6) $$\begin{array}{l} P\left(z_{m-1,i}|z_{m,n},o_{m,n,1},o_{m,n,2},\cdots \;,z_{m-1}\right). \end{array}$$
3. The module can determine *z*_*m, n*_ by using the following probability sent from module (*m* + 1, *j*) by one of the methods explained in the next section:
  (7) $$\begin{array}{l} P\left(z_{m,n}|z_{m+1,j},o_{m+1,j,1},o_{m+1,j,2},\cdots \;,z_{m}\right). \end{array}$$
4. Terminal modules have no shared latent variables and only have observations.

In Serket, the modules affecting each other and the shared latent variables are determined by their communication with each other. Methods to determine the latent variables are classified into two types depending on their nature. One is the case that they are discrete and finite, and another is the case that they are continuous or infinite.

### 3.2. Inference of composed models

In this section, we explain the parameter inference methods used for the composed models. We focus on the batch algorithm for parameter inference, which makes it easy to implement each module. Therefore, real-time application is beyond the scope of this paper although we would like to realize it in the future. One of the inference methods used to estimate the parameters of complex models is based on variational Bayesian (VB) approximation (Minka and Lafferty, 2002; Blei et al., 2003; Kim et al., 2013). However, a VB-based approach requires derivation against latent variables, and it is difficult to implement derivation in independent modules. To this end, we employed a sampling-based method because of its simpler implementation.

In this section, we utilize three approaches according to the nature of the latent variables.

#### 3.2.1. Message passing approach

**Figure 5:**
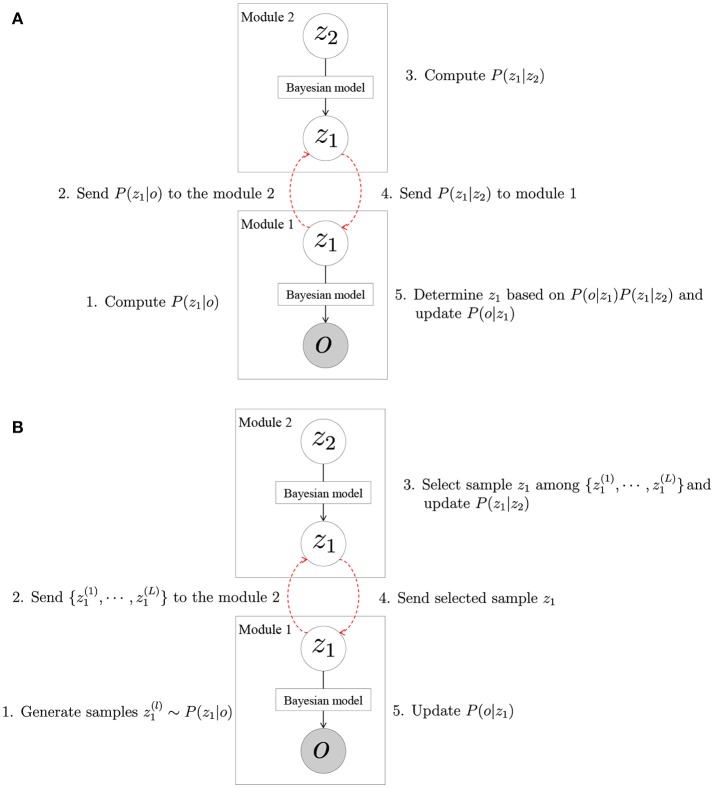
Connecting two modules by **(A)** MP approach and **(B)** SIR approach.

First, we consider the case when the latent variables are discrete and finite. For example, in the model shown in Figure 4A, the shared latent variable *z*^*O*^ was generated from a multinomial distribution, which is represented by finite dimensional parameters. Here, we consider the estimation of the latent variables according to the simplified model shown in Figure 5A. In module 2, the shared latent variable *z*_1_ was generated from *z*_2_; and in module 1, the observation *o* was generated from *z*_1_. The latent variable *z*_1_ is shared in modules 1 and 2, and determined by the effect on these two modules as follows:

(8) $$\begin{array}{l} z_{1}\sim P\left(z_{1}|o,z_{2}\right) \end{array}$$

(9) $$\begin{array}{l} \propto P\left(z_{1}|o\right)P\left(z_{1}|z_{2}\right). \end{array}$$

In this equation, *P*(***o***|*z*_1_) and *P*(*z*_1_|*z*_2_) can be computed in modules 1 and 2, respectively. We assumed that the latent variable is discrete and finite, and *P*(*z*_1_|*z*_2_) is a multinomial distribution that can be represented by a finite-dimensional parameter whose dimension ranges from the number of elements of *z*_1_. Therefore, *P*(*z*_1_|*z*_2_) can be sent from module 2 to module 1. Moreover, *P*(*z*_1_|*z*_2_) can be learned in module 2 by using *P*(*z*_1_|***o***) sent from module 1, which is also a multinomial distribution. The parameters of these distributions can be easily sent and received, and the shared latent variable can be determined by the following procedure:

1. In module 1, *P*(*z*_1_|***o***) is computed.
2. *P*(*z*_1_|***o***) is sent to module 2.
3. In module 2, the probability distribution *P*(*z*_1_|*z*_2_), which represents the relationships between *z*_1_ and *z*_2_, is estimated using *P*(*z*_1_|***o***).
4. *P*(*z*_1_|*z*_2_) is sent to module 1.
5. In module 1, the latent variable *z*_1_ is estimated using Equation (9), and the parameters of *P*(***o***|*z*_1_) are updated.

Thus, in the case when the latent variable is infinite and discrete, the modules are learned by sending and receiving the parameters of a multinomial distribution of *z*_1_. We call this the message passing (MP) approach because the model parameters can be optimized by communicating the message.

#### 3.2.2. Sampling importance resampling approach

In the previous section, the latent variable was determined by communicating the parameters of the multinomial distributions if the latent variables are discrete and finite. Otherwise, it can be difficult to communicate the parameters. For example, the number of parameters becomes infinite if the possible values of the latent variables are infinite patterns. In the case of a complex probability distribution, it is difficult to represent it by a small number of parameters. In such cases, the model parameters are learned by approximation using sampling importance resampling (SIR). We also consider parameter estimation using the simplified model shown in Figure 5B. Here, the latent variable *z*_1_ is shared, and its possible value is either an infinite pattern or continuous. Similar to the previous section, the latent variable is determined if the following equation can be computed:

(10) $$\begin{array}{l} z_{1}\sim P\left(z_{1}|o,z_{2}\right) \end{array}$$

(11) $$\begin{array}{l} \propto P\left(z_{1}|o\right)P\left(z_{1}|z_{2}\right). \end{array}$$

However, when the value of *z*_1_ is infinite or continuous, module 2 cannot send *P*(*z*_1_|*z*_2_) to module 1. Therefore, *P*(*z*_1_|***o***) is first approximated by *L* samples {*z*^(*l*)^:*l* = 1, ⋯ , *L*}:

(12) $$\begin{array}{l} z_{1}^{\left(l\right)}\sim P\left(z_{1}|o\right). \end{array}$$

This approximation is equivalent to approximating *P*(*z*_1_|*o*) by the following $\tilde{P}\left(z_{1}|o\right)$:

(13) $$\begin{array}{l} P\left(z_{1}|o\right)\approx \tilde{P}\left(z_{1}|o\right)=\frac{1}{L}\overset{L}{\underset{l}{\sum }}\delta \left(z_{1},z_{1}^{\left(l\right)}\right), \end{array}$$

where δ(*a, b*) represents a delta function, which is 1 if *a* = *b*, and 0 otherwise. The generated samples are sent from module 1 to module 2, and a latent variable is selected among them based on *P*(*z*_1_|*z*_2_):

(14) $$\begin{array}{l} z_{1}\sim P\left(z_{1}\in \left\{z_{1}^{\left(1\right)},\cdots \;,z_{1}^{\left(L\right)}\right\}|z_{2}\right). \end{array}$$

This procedure is equivalent to sampling from the following distribution, which is an approximation of Equation (11):

(15) $$\begin{array}{l} z_{1}\sim P\left(z_{1}|z_{2}\right)\tilde{P}\left(z_{1}|o\right). \end{array}$$

Thus, the parameters of each module can be updated by the determined latent variables.

#### 3.2.3. Other approaches

We have presented two methods but these are not the only ones available for parameter estimation. There are other applicable methods to estimate parameters. For example, one of the applicable methods is the Metropolis-Hastings (MH) approach. In the MH approach, samples are generated from a proposal distribution *Q*(*z*|z^*^), where z^*^ and *z* represent the current value and generated value of latent variables, respectively. Then, they are accepted according to the acceptance probability *A*(z, z^*^):

(16) $$\begin{array}{l} A\left(z,z^{*}\right)=\min\left(1,\alpha \right) \end{array}$$

(17) $$\begin{array}{l} \alpha =\frac{P\left(z^{*}\right)Q\left(z|z^{*}\right)}{P\left(z\right)Q\left(z^{*}|z\right)}, \end{array}$$

where *P*(*z*) represents the target distribution from which the samples are generated.

The model parameters in Figure 5 can be estimated by considering *P*(*z*_1_|***o***) and *P*(*z*_1_|*z*_2_,***o***) as the proposal distribution and target distribution, respectively. *P*(*z*_1_|*z*_2_,***o***) can be transformed into

(18) $$\begin{array}{l} P\left(z_{1}|z_{2},o\right)\propto P\left(z_{1}|o\right)P\left(z_{1}|z_{2}\right)P\left(z_{2}\right). \end{array}$$

Therefore, α in Equation (16) becomes

(19) $$\begin{array}{l} \alpha =\frac{P\left(z^{*}\right)Q\left(z|z^{*}\right)}{P\left(z\right)Q\left(z^{*}|z\right)}=\frac{P\left(z_{1}^{*}|z_{2},o\right)}{P\left(z_{1}|z_{2},o\right)}\cdot \frac{P\left(z_{1}|o\right)}{P\left(z_{1}^{*}|o\right)} \end{array}$$

(20) $$\begin{array}{l} =\frac{P\left(z_{1}^{*}|o\right)P\left(z_{1}^{*}|z_{2}\right)P\left(z_{2}\right)}{P\left(z_{1}|o\right)P\left(z_{1}|z_{2}\right)P\left(z_{2}\right)}\cdot \frac{P\left(z_{1}|o\right)}{P\left(z_{1}^{*}|o\right)}=\frac{P\left(z_{1}^{*}|z_{2}\right)}{P\left(z_{1}|z_{2}\right)}, \end{array}$$

Hence, the proposal distribution *P*(*z*_1_|***o***) can be computed in module 1, and the acceptance distribution can be computed in module 2. By using this approach, the parameters can be estimated while maintaining programmatic independence. The proposed value is sent to module 2, and module 2 determines whether it is accepted or not. Then, the parameters are updated according to the accepted values.

Thus, various approaches can be utilized for parameter estimation, and it should be discussed which methods are most suitable. However, we will leave this for a future discussion because of limited space.

## 4. Example 1: multilayered MLDA

First, we show that a more complex model, mMLDA, can be constructed by combining the simpler models based on Serket. By using the mMLDA, the object categories, motion categories, and integrated categories representing the relationships between them were formed from the visual, auditory, haptic, and motion information obtained by the robot. The information obtained by the robot is detailed in Appendix 2. We compared it with the original mMLDA and an independent model, where the object and motion categories were learned independently. The original mMLDA has an upper-bound performance because any approximation is not used in it. Therefore, the purpose of this experiment is to show that Serket implementation has a comparable performance with the original mMLDA.

### 4.1. Implementation based on serket

**Figure 6:**
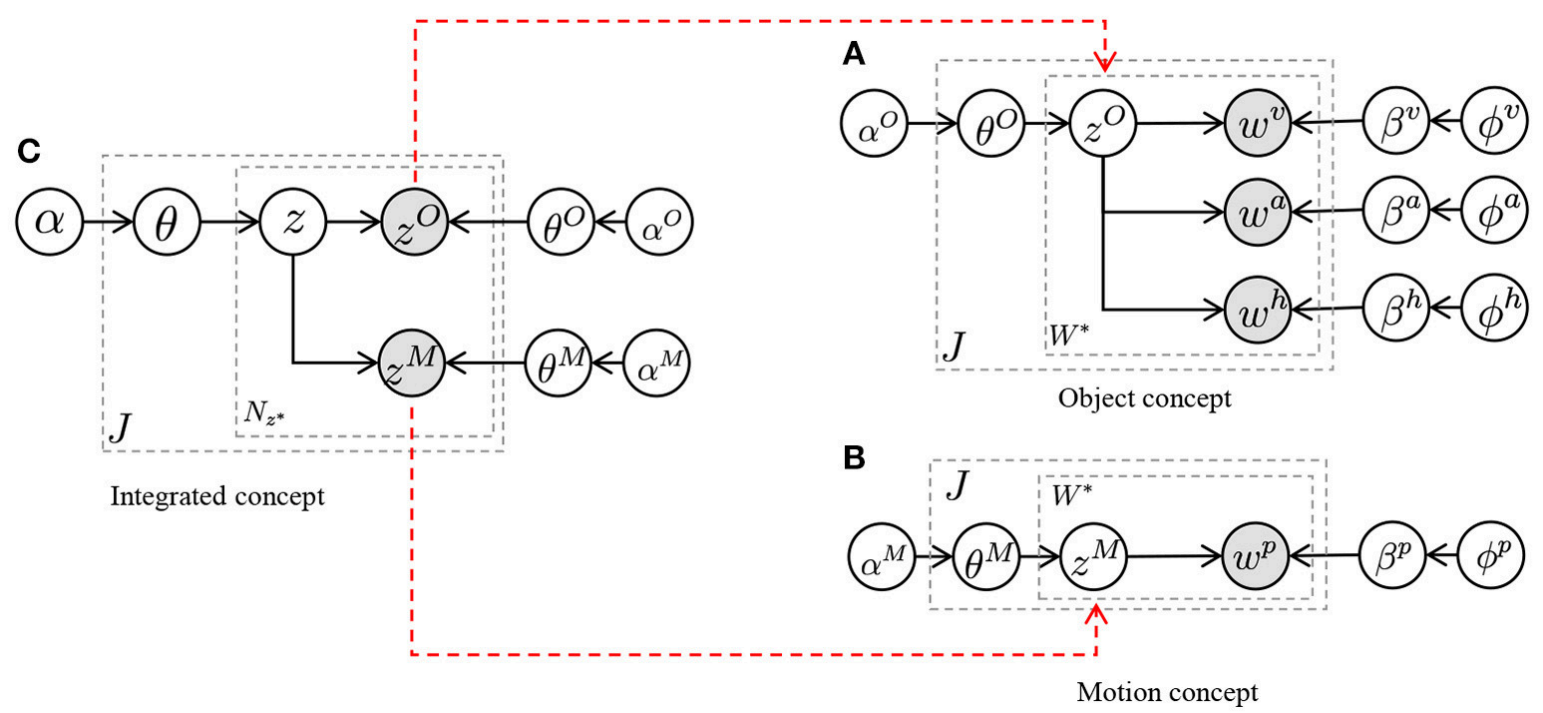
Implementation of mMLDA by connecting three MLDAs. The dashed arrows denote the conditional dependencies represented by Serket. **(A)** Object concept, **(B)** motion concept, and **(C)** integrated concept.

The mMLDA shown in Figure 4A can be constructed using the MP approach. This model can be divided into to three MLDAs. In the lower-level MLDAs, object categories *z*^*O*^ can be formed from multimodal information ***w***^*v*^, ***w***^*a*^, and ***w***^*h*^ obtained from the objects, and motion categories *z*^*M*^ can be formed from joint angles obtained by observing a human's motion. Details of the information are explained in the Appendix. Moreover, in the higher-level MLDA, integrated categories *z* that represent the relationships between objects and motions can be formed by considering *z*^*O*^ and *z*^*M*^ as observations. In this model, latent variables *z*^*O*^ and *z*^*M*^ are shared; therefore, the whole model parameters are optimized in a mutually affecting manner. Figure 6 shows the mMLDA represented by three MLDAs.

First, in the two MLDAs shown in Figures 6A,B, the probabilities $P\left(z_{j}^{O}|w_{j}^{v},w_{j}^{a},w_{j}^{h}\right)$ and $P\left(z_{j}^{M}|w_{j}^{p}\right)$ that the object and motion category of the multimodal information in the *j*-th data become $z_{j}^{O}$ and $z_{j}^{M}$, respectively, can be computed using Gibbs sampling. These probabilities are represented by finite and discrete parameters, which can be sent to the integrated concept model shown in Figure 6C, where ${ẑ}_{j}^{O}$ and ${ẑ}_{j}^{M}$ can be treated as observed variables using these probabilities.

(21) $$\begin{array}{l} {ẑ}_{jn}^{O}\sim P\left(z_{j}^{O}|w_{j}^{v},w_{j}^{a},w_{j}^{h}\right), \end{array}$$

(22) $$\begin{array}{l} {ẑ}_{jn}^{M}\sim P\left(z_{j}^{M}|w_{j}^{p}\right). \end{array}$$

where $w_{j}^{v},w_{j}^{a},w_{j}^{h}$, and $w_{j}^{p}$ represent the visual information, auditory information, haptic information, and joint angles of the human's motion, respectively, which are included in the *j*-th data.

Thus, in the integrated concept model, category *z* can be formed in an unsupervised manner. Next, the values of the shared latent variables are inferred stochastically using a learned integrated concept model:

(23) $$\begin{array}{l} P\left(z^{O}|{\hat{z}}_{j}^{M},{\hat{z}}_{j}^{O}\right)=\underset{z}{\sum }P\left(z^{O}|z\right)P\left(z|{\hat{z}}_{j}^{m},{\hat{z}}_{j}^{o}\right), \end{array}$$

(24) $$\begin{array}{l} P\left(z^{M}|{\hat{z}}_{j}^{M},{\hat{z}}_{j}^{O}\right)=\underset{z}{\sum }P\left(z^{M}|z\right)P\left(z|{\hat{z}}_{j}^{m},{\hat{z}}_{j}^{o}\right). \end{array}$$

These probabilities are also represented by finite and discrete parameters, which can be communicated using the MP approach. These parameters are sent to an object concept model and motion concept model, respectively, where the latent variables assigned to the modality information *m* ∈ {*v, a, h, p*} of concept *C* ∈ {*O, M*} are determined using Gibbs sampling.

(25) $$\begin{array}{l} z_{jmn}^{C}\sim P\left(z^{C}|W^{m},Z_{-jmn}\right)P\left(z^{C}|{\hat{z}}_{j}^{M},{\hat{z}}_{j}^{O}\right), \end{array}$$

where ***W***^*m*^ represents all the information of modality *m*, and ***Z***_−*jmn*_ represents a set of latent variables, except for the latent variable assigned to the information of modality *m* of the *j*-th observation. Whereas the latent variables were sampled from $P\left(z^{C}|W^{m},Z_{-jmn}\right)$ in the normal MLDA, they were also sampled using $P\left(z^{C}|{\hat{z}}_{j}^{M},{\hat{z}}_{j}^{O}\right)$. Therefore, all the latent variables were learned in a complementary manner. From the sampled variables, the parameters of $P\left(z_{j}^{o}|w_{j}^{v},w_{j}^{a},w_{j}^{h}\right)$ and $P\left(z_{j}^{m}|w_{j}^{m}\right)$ were updated, and Equations (21–25) were iterated until they converged.

**Figure 7:**
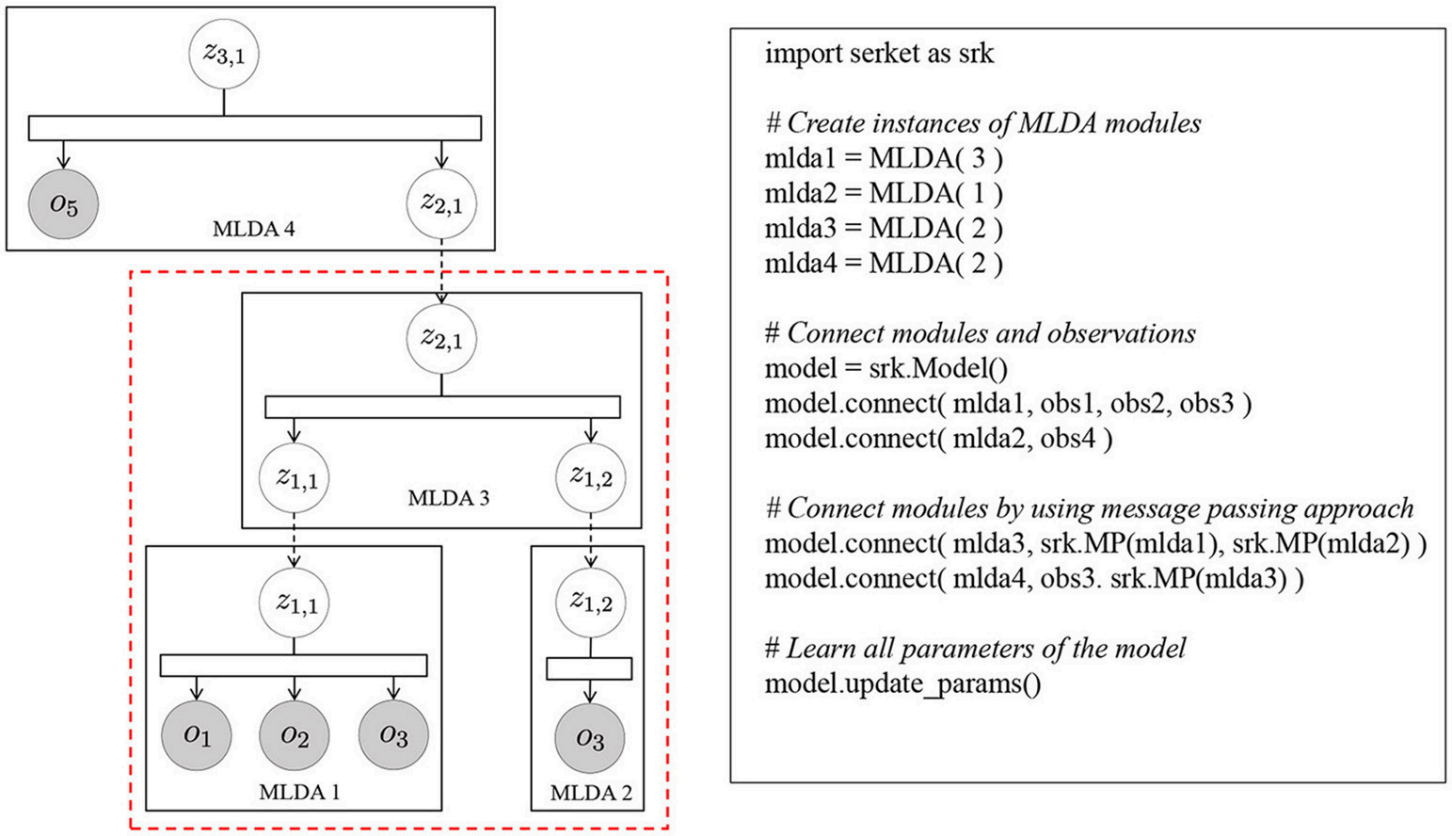
Pseudo code of mMLDA.

Figure 7 shows the pseudocode of mMLDA and the corresponding graphical model. The model on the left in Figure 7 can be constructed by connecting the latent variables based on Serket. Although the part framed by the red rectangle was implemented in the experiment, it can be easily extended to the model shown in this figure.

### 4.2. Experimental results

**Figure 8:**
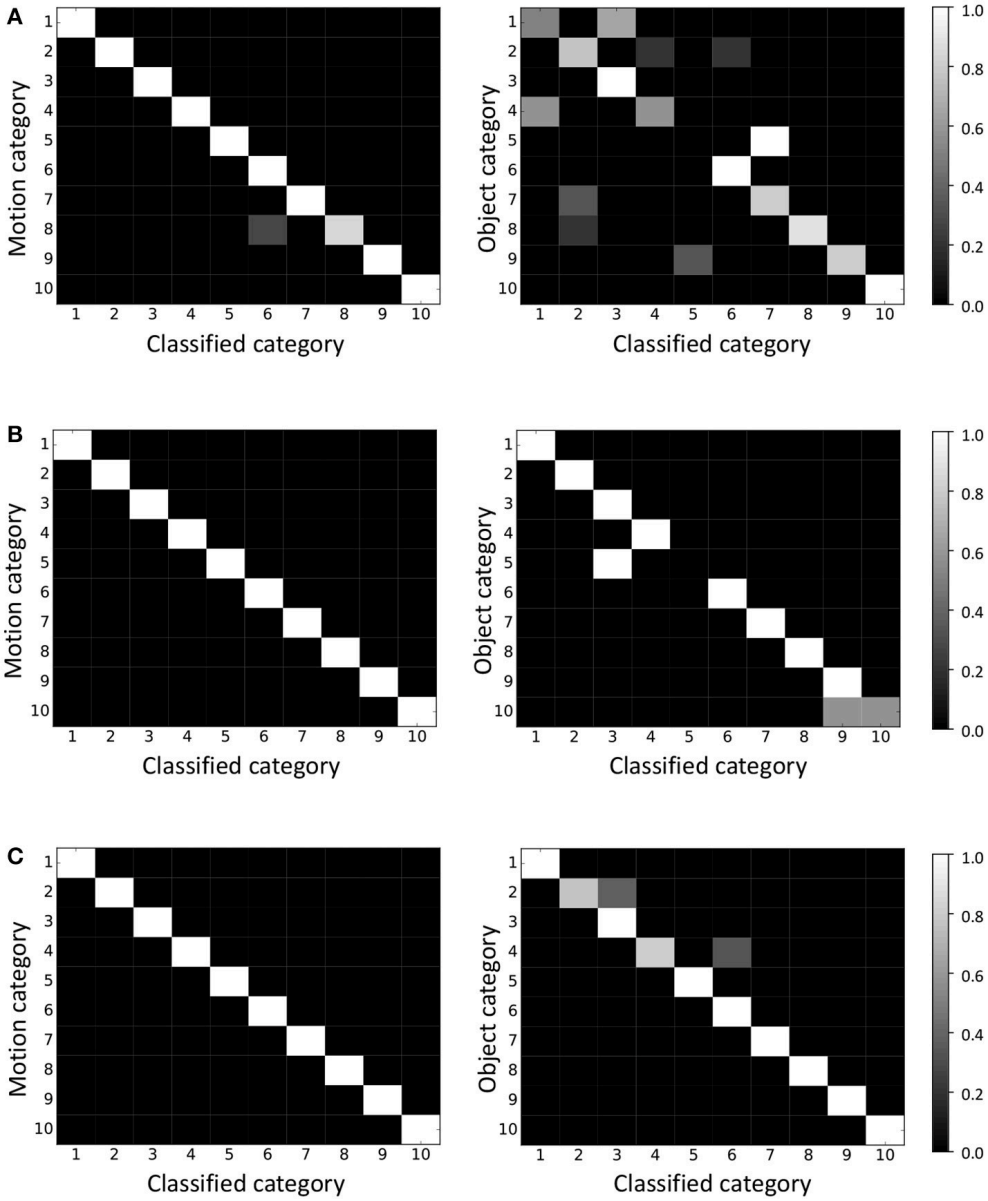
Classification results of motion and object by **(A)** independent model, **(B)** Serket implementation, and **(C)** original model. The classification accuracies for motions and objects were **(A)** 98 and 72%, **(B)** 100 and 94%, and **(C)** 100 and 94%, respectively.

Figure 8A shows a confusion matrix of classification by the model, where the object and motion categories were learned independently, and the vertical and horizontal axes represent the correct category index and the category index to which each object was classified, respectively. The accuracies were 98 and 72%. One can see that the motion categories can be formed by the independent model almost correctly. However, the object categories could not be formed correctly compared to the motion categories. On the other hand, Figure 8B shows the results of using mMLDA implemented based on Serket, and the categories were learned in a complementary manner. The classification accuracies were 100% and 94%. The motion that could not be classified correctly by the independent model was classified correctly. Moreover, the object classification accuracy improved by 22% owing to the effects of motion categories. In the independent model, category five (shampoos) objects were classified as category seven because of their visual similarity. On the other hand, in the mMLDA based on Serket, they were misclassified as category three (dressings) because the same motion (pouring) was performed with these objects. Also, the rattles (category 10) were misclassified because the rattles (category 10) and soft toys (category nine) had a similar appearance and the same motion (throwing) was performed with them. However, other objects were classified correctly, and this fact indicates that mutual learning was realized by Serket.

Furthermore, we conducted an experiment to investigate the efficiency of the original mMLDA which was not divided into modules. The results in Figure 8C show that the accuracies of the classification of objects and motions were 100 and 94%, respectively, although misclassified objects differed from that of the Serket implementation of mMLDA because of sampling. One can see that mMLDA implementation based on Serket is comparable with the original mMLDA.

Table 1 shows the computation time of mMLDA implemented by each method. The Independent model was fastest because the parameters of two MLDAs were independently learned. Serket implementation was slower than the independent model but faster than the original mMLDA. In the original MLDA, all the observations were used for parameter estimation of the integrated concept model. On the other hand, in the Serket implementation, this was approximated and only the parameters sent from lower-level MLDA in Equations (21, 22) were used for parameter estimation of the integrated concept models. Thus, the Serket implementation is faster than the original mMLDA.

**Table 1:** Computational time of mMLDA.

**Methods**	**Time (seconds)**
Independent model	1.77
Serket implementation	21.4
Original model	64.1

### 4.3. Deeper model

**Figure 9:**
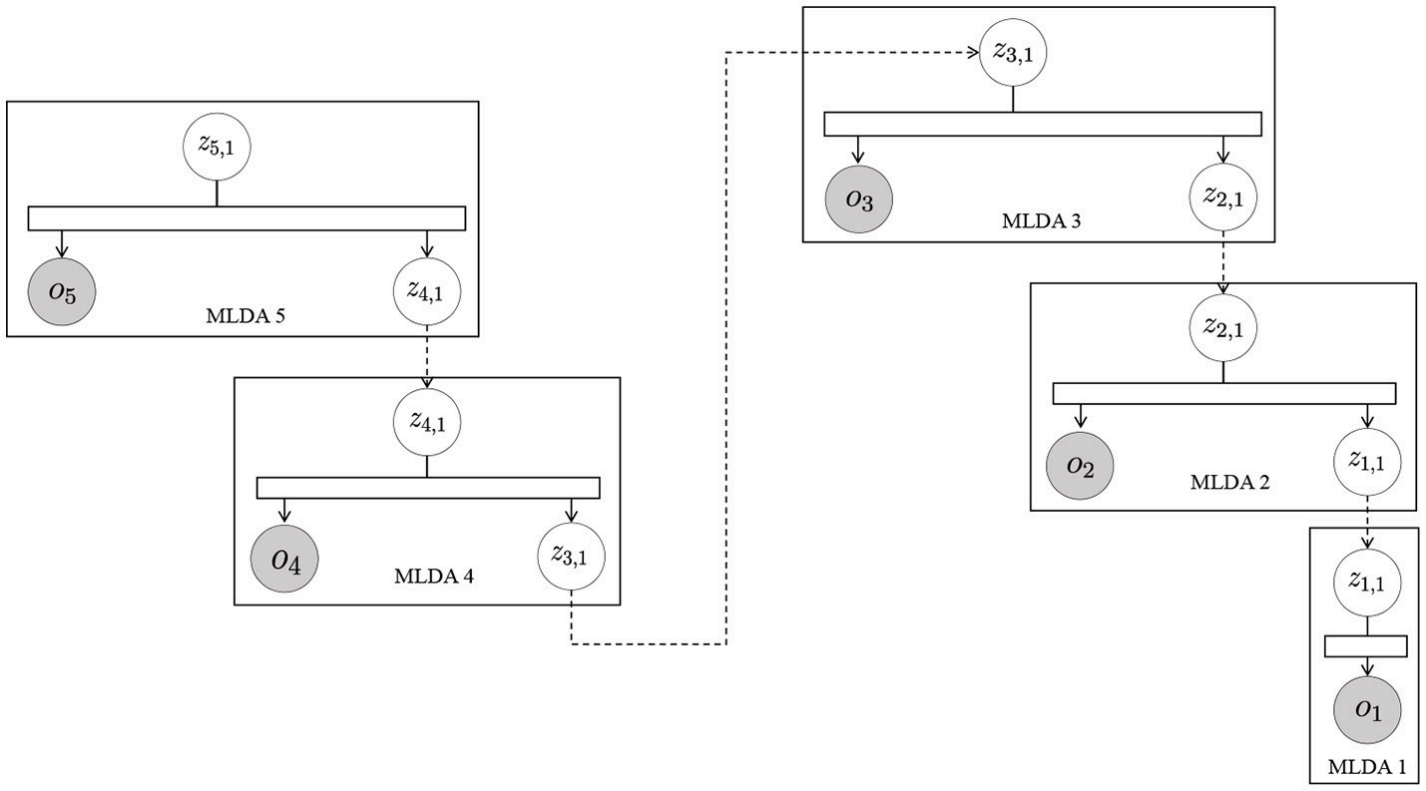
mMLDA that has five hierarchies.

In the original mMLDA, the structure of the model was fixed, and we derived the equations to estimate its parameters and then implemented them. However, by using Serket, we can flexibly change the structure of the model without deriving the equations for the parameter estimation. As one example, we changed the structure of mMLDA and constructed a deeper model as shown in Figure 9. To confirm that the parameters can be learned by using Serket, we generated training data by using the following generative process:

(26) $$\begin{array}{l} z_{5,1}\sim P\left(z|\theta_{5}\right) \end{array}$$

(27) $$\begin{array}{l} o_{5}\sim P\left(o|\phi_{z_{5,1}}\right) \end{array}$$

$$\begin{array}{l} \text{for }m=4\text{ to }1: \end{array}$$

(28) $$\begin{array}{l} \;z_{m,1}\sim P\left(z|z_{m+1,1},\theta_{m}\right) \end{array}$$

(29) $$\begin{array}{l} \;o_{m}\sim P\left(o|\phi_{z_{m,1}}\right) \end{array}$$

where *m* denote the index of hierarchies, and the number of categories of all modules was 10. ***θ***_*m*_ and ***ϕ***_*z*_ were randomly generated, and we used uniform distribution as *P*(*z*|θ_5_). This generative process was repeated 50 times, and 250 observations were made. The parameters were estimated by classifying these 250 observations through a Serket implementation and independent model. Table 2 shows the classification accuracies in each hierarchy. We can see that the Serket implementation outperformed the independent model because the parameters were optimized as a whole by using an MP approach. Usually, the equations for parameter estimation must be derived for each model individually; deriving them for a more complicated model is difficult. However, Serket makes it possible to construct a complicated model flexibly and to estimate the parameters easily.

**Table 2:** Classification accuracies of mMLDA having five hierarchies.

**Methods**	***z*_1, 1_ (%)**	***z*_2, 1_(%)**	***z*_3, 1_(%)**	***z*_4, 1_(%)**	***z*_5, 1_(%)**	**Average**
Independent model	70.0	66.0	74.0	76.0	66.0	70.4
Serket implementation	100	90.0	100	100	100	98.0

## 5. Example 2: mutual learning of concept model and language model

In Nakamura et al. (2014) and Nishihara et al. (2017), we proposed a model for the mutual learning of concepts and the language model shown in Figure 4B; its parameters were estimated by dividing the models into smaller parts. In this section, we show that this model can be constructed by Serket. To learn the model, the visual, auditory, and haptic information obtained by the robot and teaching utterances given by a human user were used. The details are explained in Appendix 2. As in the previous experiment, the original model has upper-bound performance. Therefore, the purpose of this experiment is also to show that Serket implementation has comparable performance with the original model.

### 5.1. Implementation based on serket

**Figure 10:**
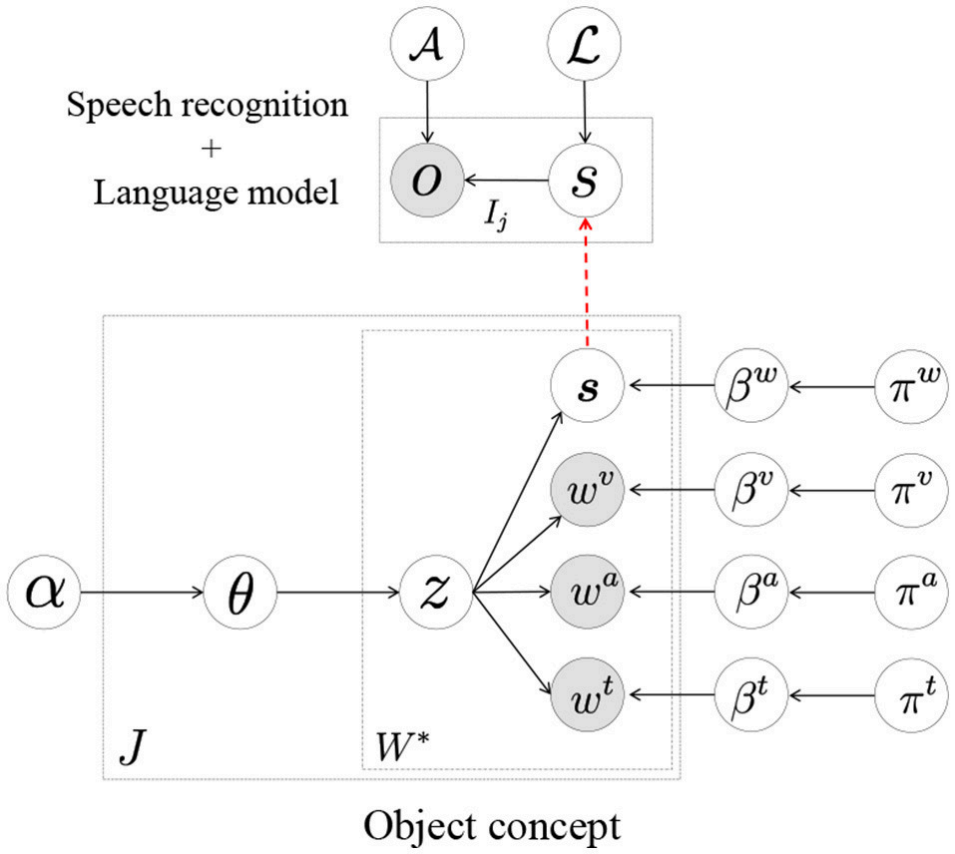
Mutual learning model of concepts and language model.

Here, we reconsider the mutual learning model based on Serket. The model shown in Figure 4B is a one where the speech recognition part and the MLDA that represents the object concepts are connected, and can be divided as shown in Figure 10. The MLDA makes it possible to form object categories by classifying the visual, auditory, and haptic information obtained, as shown in the Appendix 2. In addition, the words in the recognized strings of a user's utterances to teach object features are also classified in the model shown in Figure 10. Through this categorization of multimodal information and teaching utterance, the words and multimodal information are connected stochastically, which enables the robot to infer the sensory information represented by the words. However, the robot cannot obtain the recognized strings directly; it can only obtain continuous speech. Therefore, in the model shown in Figure 10, the words *s* which are in the recognized strings are treated as latent variables and connected to the model for speech recognition. The parameter L of the language model is also a latent variable, and is learned from the recognized strings of continuous speech ***o*** using the nested Pitman–Yor language model (NPYLM) (Mochihashi et al., 2009). Furthermore, it is an important point of this model that the MLDA and speech recognition model are connected through the words *s*, which makes it possible to learn them in a complementary manner. That is, the speech is not only recognized based on the similarity of ***o*** but is accurately recognized by utilizing the inferred words *s* from the multimodal information perceived by the robot.

First, as the initial parameter of L, we used the language model where all phonemes were generated with equal probabilities. The MP approach can be used if all teaching utterances ***O*** are recognized by using a language model whose parameter is L and the probability P(S|O,A,L) that the word sequences ***S*** are generated can be computed. However, it is actually difficult to compute the probabilities for all possible word segmentation patterns of all possible recognized strings. Therefore, we approximated this probability distribution using the SIR approach. The *L*-best speech recognition results were utilized as samples because it is difficult to compute the probabilities for all possible recognized strings. $s_{j}^{\left(l\right)}$ represents the *l*-th recognized string of a teaching utterance given the *j*-th object. By applying the NPYLM and segmenting them into words, the word sequences $S=\left\{s_{j}^{\left(l\right)}|1\leq l\leq L,1\leq j\leq J\right\}$ can be obtained.

(30) $$\begin{array}{l} S\sim P\left(S|S^{\prime },L\right). \end{array}$$

These generated samples are sent to the MLDA module, and the samples that are likely to represent multimodal information are sampled based on the MLDA whose current parameter is **Θ**:

(31) $$\begin{array}{l} \hat{s_{j}}\sim P\left(s_{j}^{\left(l\right)}|w_{j}^{v},w_{j}^{a},w_{j}^{t},\Theta \right). \end{array}$$

The selected samples $\hat{s_{j}}$ are considered as words that can represent multimodal information. Then, the MLDA parameters are updated using a set of these words $\hat{S}=\left\{\hat{s_{j}}|1\leq j\leq J\right\}$ and a set of multimodal information ***W***^*v*^,***W***^*a*^,***W***^*t*^ by utilizing Gibbs sampling.

(32) $$\begin{array}{l} \Theta =\text{argmax }P\left(\hat{S},W^{v},W^{a},W^{t}|\Theta \right). \end{array}$$

Moreover, $\hat{S}$ is sent to the speech recognition model, and the parameter L of the language model is updated.

(33) $$\begin{array}{l} L=\text{argmax }P\left(\hat{S}|{\hat{S}}^{\prime },L\right), \end{array}$$

where ${\hat{S}}^{\prime }$ denotes strings obtained by connecting words in $\hat{S}$. The parameters of the whole model can be optimized by iteration through the following process: the sampling words using Equation (30), the resampling words using Equation (31), and the updating parameters using Equations (32, 33).

**Figure 11:**
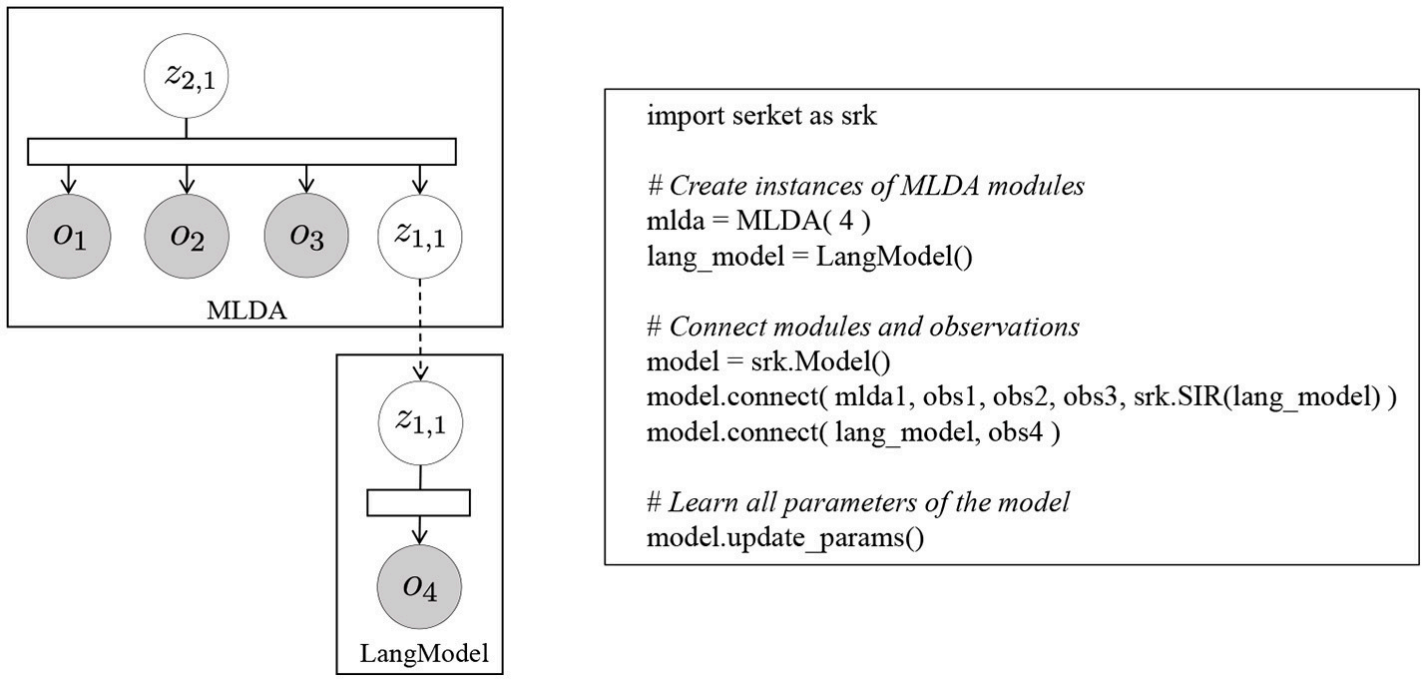
Pseudocode of mutual learning of concept model and language model.

Figure 11 displays the pseudocode and the corresponding graphical model. In this model, one of modules is MLDA with three observations and one shared latent variable connected to the speech recognition module. *o*_1_, *o*_2_, and *o*_3_ represent multimodal information obtained by the sensors on the robot, and *o*_4_, which is an observation of the speech recognition model, represents the utterances given by the human user. Although the parameter estimation of the original model proposed in Nakamura et al. (2014) and Nishihara et al. (2017) is very complicated, it can be briefly described by connecting the modules based on Serket.

### 5.2. Experimental results

We conducted an experiment where the concepts were formed using the aforementioned model to demonstrate the validity of Serket. We compared the following three methods.

(a) A method where speech recognition results $S_{0}^{\prime }$ of teaching utterances with maximum likelihoods are segmented into words by the applied NPYLM, and the words obtained are used for concept formation.
(b) A method where the concepts and language model are learned by a mutual learning model implemented based on Serket. (Proposed method)
(c) A method where the concepts and language model are learned by a mutual learning model implemented without Serket proposed in (Nakamura et al., 2014). (Original method)

In method (a), the following equation was used instead of Equation (30), and the parameter L of the language model was not updated:

(34) $$\begin{array}{l} S_{0}\sim P\left(S|S_{0}^{\prime },L\right). \end{array}$$

Alternatively, method (b) was implemented by Serket, and the concepts and language model were learned mutually through the shared latent variable ***s***.

Table 3i shows the speech recognition accuracies of each method. In method (a), the language model was not updated; therefore, the accuracy is equal to phoneme recognition. In contrast, in method (b), the accuracy is higher than that of method (a) by updating the language model from the words sampled by MLDA.

**Table 3:** Accuracies of speech recognition, segmentation, and object classification.

	**(i) Speech recognition**	**(ii) Segmentation**			**(iii) Object classification**
**Methods**		**Precision**	**Rcall**	**F-measure**	
(a) w/o mutual learning	0.64	0.50	0.68	0.58	0.80
(b) Serket implementation	0.74	0.91	0.59	0.72	0.94
(c) Original model	0.77	0.95	0.59	0.73	0.94

Table 3ii shows the accuracies of word segmentation. Segmentation points were evaluated, as shown in Table 4, by applying dynamic-programming matching to find the correspondence between the correct and estimated segmentation. This table shows a case where the correct segmentation of a correctly recognized string “ABCD” is “A/BC/D,” and the recognized string “AACD” is segmented into “A/A/CD.” (“/” represents the cut points between each word.) The points that were correctly estimated (Table 4b), as cut points were evaluated as true positive (TP), and those that were incorrectly estimated (Table 4d) were evaluated as false positive (FP). Similarly, the points that were erroneously estimated as not cut points (Table 4f) were evaluated as false negative (FN). From the evaluation of the cut points, the precision, recall, and F-measure are computed as follows.

(35) $$\begin{array}{l} P=\frac{N_{TP}}{N_{TP}+N_{FP}}, \end{array}$$

(36) $$\begin{array}{l} R=\frac{N_{TP}}{N_{TP}+N_{FN}}, \end{array}$$

(37) $$\begin{array}{l} F=\frac{2RP}{R+P}, \end{array}$$

where *N*_*TP*_, *N*_*FP*_, and *N*_*FN*_ denote the number of points evaluated as TP, FP, and FN, respectively. Comparing the precision of methods (a) and (b) in Table 3ii, one can see that it increases according to Serket. This is because more correct words can be selected among the samples generated by the speech recognition module. Alternatively, the recall of method (b) decreases because some functional words (e.g., “is” and “of”) are connected with other words such as “bottleof.” However, the precision of method (b) is higher, and its F-measure is greater than 0.11. Therefore, method (b), which was implemented based on Serket, outperformed method (a). Table 3iii displays the object classification accuracy. One can observe that the accuracy of method (b) is higher because the speech can be recognized more correctly. Moreover, the Serket implementation [method (b)] was comparable to the original implementation [method (c)]. Thus, the learning of the object concepts and language model presented in Nakamura et al. (2014); Nishihara et al. (2017) was realized by Serket.

**Table 4:** Evaluation of segmentation.

	**(a)**	**(b)**	**(c)**	**(d)**	**(e)**	**(f)**	**(g)**
Correct segmentation:	A	/	B		C	/	D
Estimated segmentation:	A	/	A	/	C		D
Evaluation:	TN	TP	TN	FP	TN	FN	TN

Table 5 shows the computation time of mutual learning models. From this figure, the model without mutual learning is fastest because the parameters of one MLDA and language model are independently learned once. On the other hand, Serket implementation is slower and comparable with the original model. This is because the parameters of the MLDA and language model in the Serket implementation were updated iteratively by communicating the parameters with the MP approach, and the computational cost was not much different from that of the original model.

**Table 5:** Computation time of mutual learning model.

**Methods**	**Time (seconds)**
w/o mutual learning	135
Serket implementation	2,640
Original model	2,637

## 6. Conclusion

In this paper, we proposed a novel architecture where the cognitive model can be constructed by connecting modules, each of which maintains programmatic independence. Two approaches were used to connect these modules. One is the MP approach, where the parameters of the distribution are of a finite dimension and communicated between the modules. If the parameters of the distribution are of an infinite dimension or a complex structure, the SIR approach was utilized to approximate them. In the experiment, we demonstrated two implementations based on Serket and their efficiency. The experimental results demonstrated that the implementations are comparable with the original model.

However, there is an issue with regard to the convergence of the parameters. If a large number of samples can be obtained, each latent variable can be locally converged into global optima because the MP, SIR, and MH approaches are based on the existing Markov chain Monte Carlo method. But when various types of models are connected, it is not clear whether all latent parameters can be converged into global optima as a whole. It was confirmed that the parameters were converged in the models used in the experiments. Nonetheless, this remains a difficult and important issue which will be examined in future work.

We believe that models that can be connected by Serket are not limited to generative probabilistic models, although we focused on the connected generative probabilistic models in this paper. Neural networks or other methods can be one of the modules of Serket, and we are planning to connect them. Furthermore, we believe that large-scale cognitive models can be constructed by connecting various types of modules, each of which represent a particular brain function. In so doing, we will realize our goal of artificial general intelligence. Serket can also contribute to developmental robotics (Asada et al., 2009; Cangelosi et al., 2015), where the human developmental mechanism is understood via a constructive approach. We believe that robots can learn capabilities ranging from motor skills to language, and these can be developed using Serket, as it makes it possible to understand humans.

## Author contributions

ToN, TaN and TT conceived of the presented idea. ToN developed the theory and performed the computations. ToN wrote the manuscript with support from TaN and TT. TaN and TT supervised the project. All authors discussed the results and contributed to the final manuscript.

### Conflict of interest statement

The authors declare that the research was conducted in the absence of any commercial or financial relationships that could be construed as a potential conflict of interest.

## Footnotes

^1^Symbol emergence in robotics focuses on the real and noisy environment, and the *e* in Serk*e*t represents a false recognition obtained through learning in such an environment.
