# Supplementary material for: SERKET: An Architecture for Connecting Stochastic Models to Realize a Large-Scale Cognitive Model
Source: Front Neurorobot. 2018 Jun 26;12:25. doi: 10.3389/fnbot.2018.00025 (PMC6028621; doi:10.3389/fnbot.2018.00025)
Supplement: Supplementary file 1 [file Data_Sheet_1.pdf]

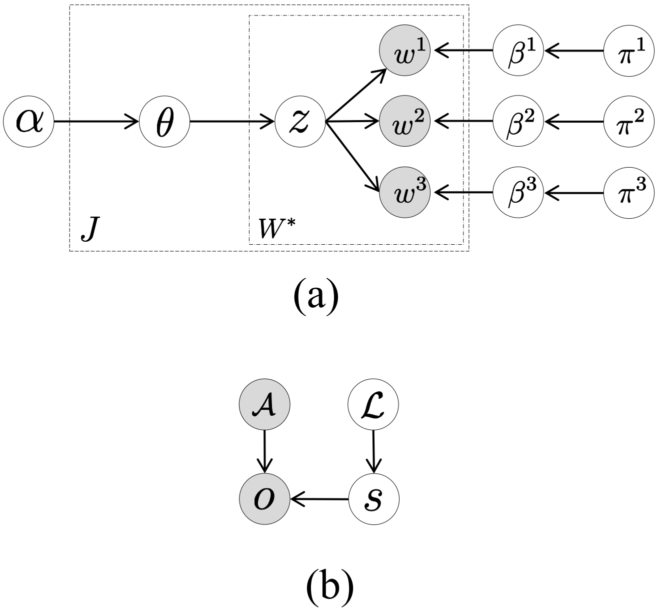

Figure 1. (a) Model for concept formation, and (b) model for learning of the language model.

APPENDIX 1: FUNDAMENTAL MODELS

In this section, we detail the multimodal latent Dirichlet allocation (MLDA) and learning of the language model, which were used in two applications in this paper.

Multimodal LDA

We used MLDA as one of the modules. MLDA is an extension of LDA (Blei et al., 2003), which was proposed for document classification, to classify multimodal information obtained by the robot's sensors. In this model, it was assumed that the multimodal information w_1, w_2 and w_3 was generated by following generative process:

- Category ratio is determined:

$$\theta \sim P(\theta|\beta). \tag{1}$$

- Following process is iterated N_m times for $m \in \{1, 2, 3\}$:
  1. A category is selected:

$$z \sim P(z|\theta). \tag{2}$$

  2. Information of category z is generated:

$$w_m \sim P(w|\phi_{mz}). \tag{3}$$

MLDA stochastically models the generative process of multimodal information, and multimodal information w_m was assumed to be sampled from the distribution $P(w_m|\phi_{mz})$, such that information w_m of category z of modality m is generated. Fig. 1(a) is a graphical model of MLDA, and depicts

this generative process of multimodal information. As well as LDA, the categories can be learned in an unsupervised manner using Gibbs sampling, where category z is sampled and the model parameters θ, ϕ_m are estimated. MLDA is the most fundamental model for multimodal categorization, and it can be extended to the multimodal hierarchical Dirichlet process (Nakamura et al., 2011), which makes it possible to estimate the number of categories, as well as an infinite mixture of models (Nakamura et al., 2015), which makes it possible to estimate the model structure.

In terms of multimodal information, we used visual, auditory, and haptic information, which we will explain later. In the previous study (Nakamura et al., 2007), we indicated that more human-like categories can be formed by the classification of multimodal information.

Learning of language model

The robot can form object concepts using MLDA, and acquire word meanings by connecting the formed concepts and words learned through interaction with others. To obtain the words, the robots are required to recognize speech and extract words from it. In order to do so, a language model is required, which can be learned in an unsupervised manner using the model shown in Fig. 1(b). The variable o represents the given human speech, and this is recognized and converted into a sequence of words s using the parameters of the acoustic model $\mathcal{A}$ and language model $\mathcal{L}$. Here, we consider that $\mathcal{A}$ is already known and $\mathcal{L}$ is learned. In the initial learning phase, the parameters of the language model are unknown, and we set these to a uniform distribution where all phonemes are equally generated. First, the parameters of the language model $\mathcal{L}$ can be estimated by dividing the recognized strings into words using a nested Pitman–Yor language model (NPYLM) (Mochihashi et al., 2009), which is a method for unsupervised morphological analysis. This word segmentation was realized via an estimation parameter $\mathcal{L}$, which maximizes the probability that the word sequence $\boldsymbol{S} = \{w_1^w, w_2^w, \cdots\}$ of recognized strings $\boldsymbol{S}'$ is generated.

$$\mathcal{L}, \boldsymbol{S} = \underset{\mathcal{L}, \boldsymbol{S}}{\operatorname{argmax}} P(\boldsymbol{S}|\boldsymbol{S}'\mathcal{L}). \tag{4}$$

The learned language model enables the robot to recognize speech accurately.

Hierarchical Pitman-Yor Language Model

The hierarchical Pitman–Yor language model (HPYLM) is an n-gram language model in which the hierarchical Pitman–Yor process is used. In the HPYLM, the probability that a word w appears after a context h is computed as follows:

$$p(w|h) = \frac{c(w|h) - d \cdot t_{hw}}{\theta + \sum_w c(w|h)} + \frac{\theta + d \cdot \sum_w t_{hw}}{\theta + \sum_w c(w|h)} p(w|h'), \tag{5}$$

where h' represents an $(n-1)$-gram context and $p(w|h')$ is the probability that the word w appears after the context that is one shorter than h; therefore, these probabilities can be computed recursively. Also, $c(w|h)$ represents the number of occurrences of w, and t_{hw} represents the number of occurrences of w in the context h. d and θ are the hyperparameters of the Pitman–Yor process.

Nested Pitman-Yor Language Model

In the HPYLM mentioned in the previous section, $p(w|h')$ in Eq. (5) can be set as the reciprocal of the number of vocabulary in the case of unigram. However, we assumed that the vocabulary is not predefined, and is difficult to compute because all possible substrings in the recognized strings can be words. In

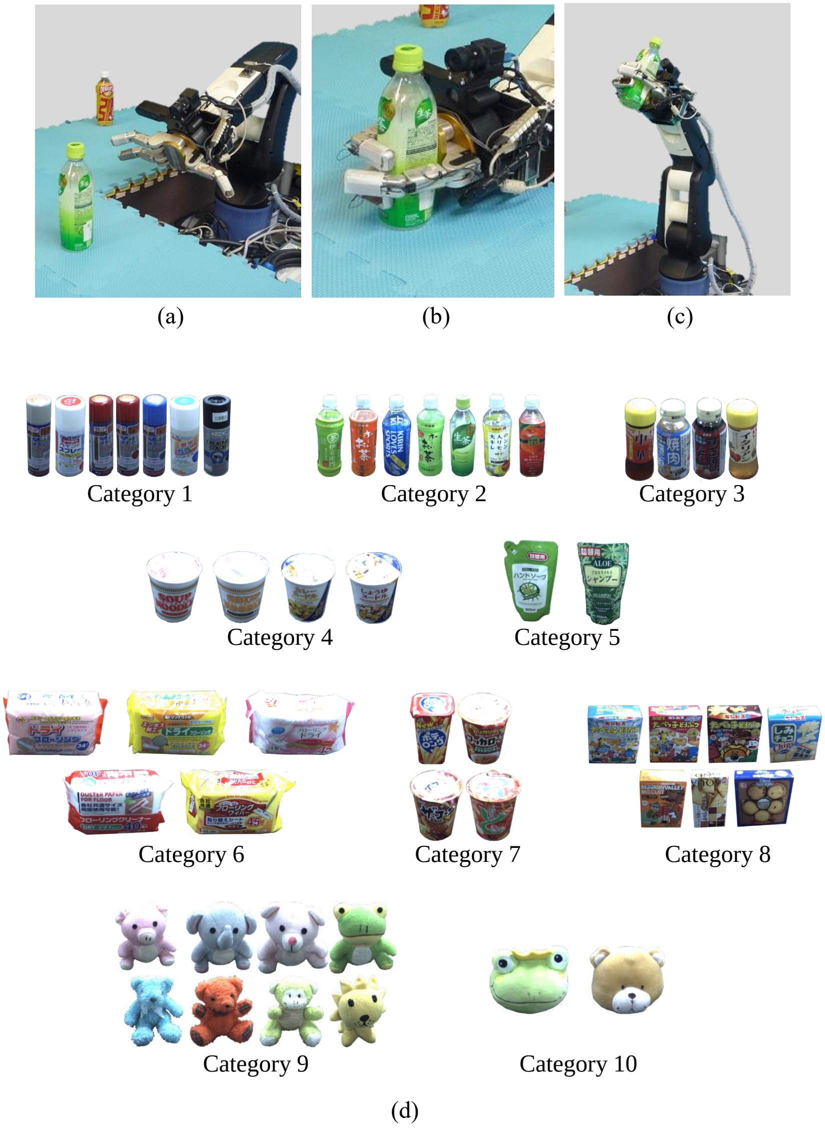

Figure 2. Obtaining (a) visual information, (b) haptic information, (c) auditory information, and (d) 50 objects used in the experiments.

order to solve this problem, the character HPYLM was used as the base measure of the word unigram. This model is called the NPYLM because the character HPYLM is embedded in the word HPYLM. By utilizing the blocked Gibbs sampler and dynamic programming, NPYLM can divide strings into words efficiently.

APPENDIX 2: MULTIMODAL OBJECT DATASET

Fig. 2(d) displays the objects used in the experiments. The robot obtained the multimodal information from these objects.

Visual information w^v A charge-coupled device camera and depth sensor were mounted on the arm of the robot (Fig. 2(a)), and the images captured by observing the objects were utilized for visual information. A dense scale-invariant feature transform (Vedaldi and Fulkerson, 2010) was computed

Table 1. Motions that are carried out against objects.

motion	object	motion	object
pour (1)	dressing	wipe (5)	flooring cleaner
	shampoo	spray (6)	spray can
shake (2)	spray can	look (7)	soft toy
	plastic bottle	put (8)	snack
	dressing		cup noodle
drink (3)	plastic bottle	throw (9)	soft toy
eat (4)	cup noodles		rattle
	snack	pick up (10)	cookie

from each image. Each feature vector was quantized using 500 representative vectors and converted into a 500-dimensional histogram.

Haptic information w^t

Haptic information was obtained using a Barrett hand mounted on the arm, and a tactile array sensor was mounted on the hand (Fig. 2(b)). The robot grasps the objects and obtains a time series of sensor values. The sensor values were approximated by a sigmoid function, the parameters of which were used as feature vectors (Araki et al., 2011). Finally, these feature vectors were quantized and converted into a 15-dimensional histogram.

Auditory information w^a

A microphone was mounted on the robot's hand, and the sound was captured by shaking the objects (Fig. 2(c)). The sound was divided into frames, and a 13-dimensional mel-frequency cepstral coefficient was computed from each frame. Therefore, the sound was converted into a 13-dimensional feature vector. As well as the other information, these feature vectors were quantized and converted into a 50-dimensional histogram.

Motion information w^p

Motion information was computed from the joint angles captured by Microsoft Kinect. The sequence of 11 joint angles was captured. We assumed that each motion can be segmented based on the identity of the manipulated object; therefore, the sequence can be considered from the beginning to the end of the manipulation of each object as one motion. Table 1 displays the motions carried out against each object. The 11 joint angles were treated as 11-dimensional feature vectors, which were quantized and converted into a 70-dimensional histogram. This histogram is a bag of feature representations of motion, the efficiency of which is shown in (Mangin and Oudeyer, 2012).

Teaching utterances o

The speech that a human user provides to teach object features was used as the teaching utterances. Each speech corresponds to each object based on the object identities. Therefore, the speech uttered during a robot's observing, grasping, and shaking is assumed to represent its object features.

In the experiment, the multimodal information was obtained through the following procedure. First, the user placed an object in front of the robot. After detecting the object, the robot observed, grasped, and shook it to obtain multimodal information. Simultaneously, the user teaches the object features by speech. We instructed the user to teach the object features and did not impose any restrictions with regard to their expression.

REFERENCES

Araki, T., Nakamura, T., Nagai, T., Funakoshi, K., Nakano, M., and Iwahashi, N. (2011). Autonomous acquisition of multimodal information for online object concept formation by a robot. In *IEEE/RSJ International Conference on Intelligent Robots and Systems*. 1540–1547

Blei, D. M., Ng, A. Y., and Jordan, M. I. (2003). Latent Dirichlet Allocation. *Journal of Machine Learning Research* 3, 993–1022

Mangin, O. and Oudeyer, P.-Y. (2012). Learning to recognize parallel combinations of human motion primitives with linguistic descriptions using non-negative matrix factorization. In *IEEE/RSJ International Conference on Intelligent Robots and Systems*. 3268–3275

Mochihashi, D., Yamada, T., and Ueda, N. (2009). Bayesian Unsupervised Word Segmentation with Nested Pitman-Yor Language Modeling. In *Joint Conference of the 47th Annual Meeting of the ACL and the 4th International Joint Conference on Natural Language Processing*. vol. 1, 100–108

Nakamura, T., Ando, Y., Nagai, T., and Kaneko, M. (2015). Concept formation by robots using an infinite mixture of models. In *IEEE/RSJ International Conference on Intelligent Robots and Systems*. 4593–4599

Nakamura, T., Nagai, T., and Iwahashi, N. (2007). Multimodal Object Categorization by a Robot. In *IEEE/RSJ International Conference on Intelligent Robots and Systems*. 2415–2420

Nakamura, T., Nagai, T., and Iwahashi, N. (2011). Multimodal categorization by hierarchical dirichlet process. In *IEEE/RSJ International Conference on Intelligent Robots and Systems*. 1520–1525

Vedaldi, A. and Fulkerson, B. (2010). VLFeat: An Open and Portable Library of Computer Vision Algorithms. In *ACM International Conference on Multimedia*. 1469–1472
